# Alterations in gray matter volume and associated transcriptomics after electroconvulsive therapy in major depressive disorder

**DOI:** 10.1017/S0033291725000868

**Published:** 2025-04-21

**Authors:** Xiaoxue Liu, Jinpeng Niu, Wei Liao, Lian Du

**Affiliations:** 1 Department of Psychiatry, Key Laboratory of Major Brain Disease and Aging Research (Ministry of Education), The First Affiliated Hospital of Chongqing Medical University; 2 Key Laboratory of Major Brain Disease and Aging Research (Ministry of Education), Chongqing Medical University, Chongqing, China; 3 MOE Key Lab for Neuroinformation, High-Field Magnetic Resonance Brain Imaging Key Laboratory of Sichuan Province, University of Electronic Science and Technology of China, Chengdu, P.R. China

**Keywords:** brain-wide transcriptomic, electroconvulsive therapy, gene enrichment analysis, gray matter volume, major depressive disorder

## Abstract

**Background:**

The antidepressant mechanism of electroconvulsive therapy (ECT) remains not clearly understood. This study aimed to detect the changes in gray matter volume (GMV) in patients with major depressive disorder (MDD) caused by ECT and exploratorily analyzed the potential functional mechanisms.

**Methods:**

A total of 24 patients with MDD who underwent eight ECT sessions were included in the study. Clinical symptom assessments and MRI scans were conducted and compared. Using whole-brain micro-array measurements provided by the Allen Human Brain Atlas (AHBA), regional gene expression profiles were calculated. The differential gene PLS1 was obtained through Partial Least Squares (PLS) regression analysis, and PLS1 was divided into positive contribution (PLS1+) and negative contribution (PLS1−) genes. Through gene function enrichment analysis, the functional pathways and cell types of PLS1 enrichment were identified.

**Results:**

Gray matter volume (GMV) in the somatosensory and motor cortices, occipital cortex, prefrontal cortex, and insula showed an increasing trend after ECT, while GMV in the temporal cortex, posterior cingulate cortex, and orbitofrontal cortex decreased. PLS1 genes were enriched in synapse- and cell-related biological processes and cellular components (such as ‘pre- and post-synapse’, ‘synapse organization’ etc.). A large number of genes in the PLS1+ list were involved in neurons (inhibitory and excitatory), whereas PLS1− genes were significantly involved in Astrocytes (Astro) and Microglia (Micro).

**Conclusions:**

This study established a link between treatment-induced GMV changes and specific functional pathways and cell types, which suggests that ECT may exert its effects through synapse-associated functional and affect neurons and glial cells.

## Introduction

Major depressive disorder (MDD) is a common mental illness that gravely impairs patients’ physical and mental health, with high rates of morbidity, suicide, and recurrence (Wang et al., 2018). Physical therapy, psychotherapy, and pharmaceutical therapy are primary treatment methods for MDD. A traditional physical therapy approach, electroconvulsive therapy (ECT) is considered one of the fastest-acting and most effective treatment methods for MDD (Jørgensen, Rozing, Kellner, & Osler, 2020; Mulders et al., 2020), particularly for patients with high suicide risk, depressive catatonia, more serious symptoms, or treatment-resistant conditions. However, the mechanism of action of ECT remains unclear, which limits its use in clinical settings to some extent.

MDD is thought to be a disorder with abnormalities in both brain structure and function (Drevets, 1998; Soares & Mann, 1997), A growing number of neuroimaging research programs aim to find biomarkers of MDD (Elman et al., 2017; Hibar et al., 2015). Gray matter, the center of information processing, is sensitive to a variety of external stimuli. The brain’s cerebral cortex, brain stem, and spinal cord comprise the majority of the gray matter in the brain. Gray matter volume (GMV) abnormalities across multiple brain areas have been found in patients with MDD (Gryglewski et al., 2019; Joshi et al., 2016; Ota et al., 2015) and Meta-analysis (Enneking, Leehr, Dannlowski, & Redlich, 2020; Gbyl & Videbech, 2018; Kambeitz et al., 2017; Wang et al., 2017; Xu et al., 2019). According to previous research using structural magnetic resonance imaging (MRI), the frontal limbic region—which includes the dorsolateral prefrontal cortex, anterior cingulate cortex, posterior cingulate cortex/precuneus, and orbitofrontal cortex—is the main altered area in the brain structural network associated with MDD (Janiri et al., 2020; Liao et al., 2011; Serra-Blasco et al., 2021; Sha, Wager, Mechelli, & He, 2019). A global collaborative study involving 328 patients reported that almost all brain regions showed a statistically significant increase in volume following ECT, with the exception of the cerebellum (Ousdal et al., 2020). Animal experiments further indicated that ECT could induce neurogenesis, gliogenesis, angiogenesis, and synaptogenesis (Hellsten et al., 2005; Wennström et al., 2006; Wennström, Hellsten, & Tingström, 2004), which could help us better understand the structural effects of ECT on the brain. However, we cannot directly determine the underlying mechanisms of the brain structure changes caused by ECT from human studies.

Recently, whole-brain gene expression profiles connect the transcriptome and neuroimaging (Fornito, Arnatkevičiūtė, & Fulcher, 2019). Six donated postmortem brains were used to create the Allen Human Brain Atlas (AHBA, http://www.human.brain-map.org) microarray dataset, which includes expression levels of over 20,000 genes encompassing the entire brain. This offers a roundabout way to investigate the correlation between changes in macroscopic neuroimaging phenotypes and the spatial distribution of gene expression, which has been effectively employed to link whole-brain transcriptomics with neuroimaging data for different psychiatric disorders (Arnatkeviciute, Fulcher, & Fornito, 2019). When neuroimaging and gene transcripts are combined, it becomes clearer how disease-related microscopic changes cause macroscopic brain abnormalities in mental illnesses (Morgan et al., 2019; Romero-Garcia et al., 2020; Romero-Garcia et al., 2019; Romme et al., 2017; Seidlitz et al., 2020a). A previous study examined the colocalization of genes linked to cortical thickness in MDD patients following double-frontal ECT treatment (Ji et al., 2023), However, the mechanism for other ECT sites, such as the commonly used bilateral temporal lobe stimulation (bitemporal), remains unclear. In addition, the structural features of gray matter are typically measured by gray matter volume, density, cortical area, furrow pattern, and cortical thickness (Lv et al., 2008), thus cortical thickness cannot fully reflect the structural changes of gray matter caused by ECT.

This study further used GMV to explore bitemporal ECT-induced structural changes in patients with MDD and identified its particular functional pathways and cell pathways through partial least squares (PLS) regression analysis, gene ontology enrichment analysis, and gene set enrichment analysis (GSEA).

## Materials and method

### Participants

This is an observational clinical study that included patients with MDD who were hospitalized in the psychiatric department of the First Affiliated Hospital of Chongqing Medical University. Inclusion criteria are as follows: diagnosed with MDD according to Structured Clinical Interview for Diagnostic and Statistical Manual of Mental Disorders-IV (SCID-IV/P, Chinese version) (First, 2013); has suicidal thoughts, plans, or behaviors in recent two weeks; aged between 18 and 60 years old; scheduled for ECT by the clinician; consented to participate in this study. Patients were excluded when the following conditions are present: has a history of alcohol or drug abuse; diagnosed with other mental disorders; with neurologic or other serious somatic diseases; with morphologic anomalies in the brain; has any electronic or metal implants.

A total of 24 patients with MDD participated in this study. Disease characteristics and general demographic characteristics are shown in Table 1, all the values quantitative variable are illustrated as mean ± SD. We would collect their disease-related and MRI scan information before and after eight ECT sessions. We did not control the medication of subjects during this study, which was decided by their clinician independently. Most of them (95.83%) used antidepressants continuously before the start of ECT and throughout the entire procedure. Fifteen patients (68.18%) used selective serotonin reuptake inhibitors (SSRI), while the other patients used Noradrenergic and specific serotonergic antidepressants (NASSA) or Serotonin-norepinephrine reuptake inhibitors (SNRI). The types and doses of antidepressants were decided according to the clinician’s advice (Table 1). The study protocol was reviewed and approved by the Local Medical Ethics Committee of the First Affiliated Hospital of Chongqing Medical University.

**Table 1.** Demographics and clinical characteristics of patients

Demographics	MDD (n=24)	
Age (years)	31.54 ± 10.77	
Sex (male/female)	9/15	
Education (years)	10.96 ± 2.85	
Clinical characteristics		
Age of first onset (years)	27.54 ± 12.17	
Duration of current depressive episode (months)	2.98 ± 6.40	
	Pre-ECT	Post-ECT
Numbers of patients with antidepressants (%)	23 (95.83%)	23 (95.83%)
Numbers of types of antidepressants		
One type	17 (70.83%)	11 (45.83%)
Two types	6 (25%)	10 (41.67%)
Three types or more	0	2 (8.70%)

*Note*: The values are illustrated as mean ± SD. ECT, electroconvulsive therapy; MDD, major depressive disorder.

### Electroconvulsive therapy procedure

Patients underwent modified bitemporal ECT with a brief pulse (pulse width = 0.5 ms)/constant current Thymatron DGx apparatus (Somatics LLC, Lake Bluff, IL) at the Department of Psychiatry of the First Affiliated Hospital of Chongqing Medical University. The first 3 ECT sessions were performed consecutively, and the rest sessions were performed every other day or two days. Each patient received a minimum of eight ECT sessions, then it could be continued if clinical symptoms had not reached a level of remission based on the doctors’ advice, up to a maximum of 12 sessions. Anesthesia was administered using propofol (1.5–2 mg/kg) and succinylcholine (0.5–1 mg/kg). The initial electricity is calculated according to the age of the patient: electricity % = age × 70%, resistance < 1300 Ω. Stimulus energy was adjusted according to seizure duration. If seizure duration was <25 s, the energy was increased by 5% in the subsequent ECT session (Li et al., 2023).

### Image acquisition

Data were acquired on a 3.0 Tesla MRI system (GE Signa) at the First Affiliated Hospital of Chongqing Medical University. Three-dimensional T1-weighted anatomical images were acquired before ECT and after eight sessions (repetition time: 6.4 ms; echo time: 2.5 ms; scan time: 3 min 58 s; flip angle: 8°; readout FOV: 252 mm; phase FOV: 220 mm; slice: 180; slice thickness: 1 mm).

### Image preprocessing

The 3D T1w images were pre-processed with a standard automated pipeline in FreeSurfer v6.0 (http://surfer.nmr.mgh.harvard.edu). Specifically, the cortical surface of each participant was reconstructed as follows (Fischl, 2012): skull stripping, motion correction, classification of gray matter and white matter, coordinate transformation, separation of left and right hemispheres, and construction of gray/white interface and pial surface.

To reveal the difference in GMV pre- and post-ECT treatment, a surface parcellation was first obtained by transforming the HCP atlas (360 regions) into an individual’s surface. Then, GMV was extracted for each region. Finally, a paired *t*-test was used to compare the volume difference of each brain region pre- and post-ECT treatment, using total intracranial volume (TIV) as a covariate. To understand whether the underlying mechanisms reveal the antidepressant effects of ECT, we further investigated the relation between improved depressive symptoms and changes in GMV with a PLS regression analysis.

### Brain-wide transcriptomic data processing

Brain-wide transcriptomic data were obtained from the Allen Human Brain Atlas (AHBA) dataset, which quantified the expression levels of more than 20,000 genes (http://human.brain-map.org). Transcriptomic data were processed using the *abagen* toolbox as follows (Markello et al., 2021): (i) updating probe-to-gene annotations, probes that do not match a valid Entrez ID were discarded; (ii) excluding probes with less intensity than the background in ≥50% of samples across six donors; (iii) selecting the probe for each gene with the most consistent pattern of regional variation across donors, as quantified using differential stability (DS); (iv) mapping samples to specific parcels of HCP atlas within 2 mm Euclidean distance; (v) normalizing gene expression values for each donor with a scaled robust sigmoid to address inter-subject variation; (vi) samples assigned to the same brain region were averaged separately for each donor and then across donors. This analysis was restricted to the left hemisphere as only two donors have tissue samples collected across both the left and right hemispheres (Arnatkeviciute et al., 2019). Finally, a 180 × 15633 transcription matrix containing gene expression values for each gene in each region was obtained.

### PLS regression analysis

PLS regression analysis was performed to reveal the relationship between gene expression (predictive variable) and GMV change patterns (response variable). The first component of PLS (PLS1) is a linear combination of weighted gene expression and is mostly correlated with the change pattern of GMV. The statistical significance of the variance explained by PLS1 was assessed by permuting response variables 1000 times based on spherical rotations (https://github.com/frantisekvasa/rotate_parcellation). Then, the variability of each gene in PLS1 was evaluated by bootstrapping 1000 times, and the *Z*-score was defined as the ratio of the weight of each gene to the standard error. Finally, the *Z*-scores were corrected with FDR, resulting in two lists of genes: PLS1+ and PLS1− (*p*_FDR_ < 0.001), showed significant positive and negative correlations between their expression patterns and GMV changes.

### Gene enrichment analysis

Functional annotations of Gene Ontology (GO) gene sets were performed with Metascape (https://metascape.org/gp/index.html#/main/step1), which were used to identify specific biological processes, molecular functions, and cellular components of genes in PLS1. The enrichment pathway threshold was 0.05 and FDR correction was conducted.

To assign PLS1 genes to typical cell types, gene sets of seven cell types were obtained from Seidlitz et al., including astrocytes (Astro), endothelial cells (Endo), microglia (Micro), excitatory neurons (Neuro-Ex), inhibitory neurons (Neuro-In), oligodendrocytes (Oligo) and oligodendrocyte precursor (OPC) (Seidlitz et al., 2020a). Subsequently, a gene set enrichment analysis (GSEA) was performed to assess whether identified PLS1 genes identified in previous studies were overrepresented in the specific cell types. An enrichment score was obtained for each gene set, and the enrichment score was compared with those estimated from permutation tests (*n* = 10,000) and derived a normalized enrichment score (NES) to account for the gene set size. Finally, multiple comparisons of seven gene sets were corrected using FDR. The GCEA was performed using the clusterProfiler package (https://bioconductor.org/packages/release/bioc/html/clusterProfiler.html).

## Results

### GMV changes induced by ECT

**Figure 1.**
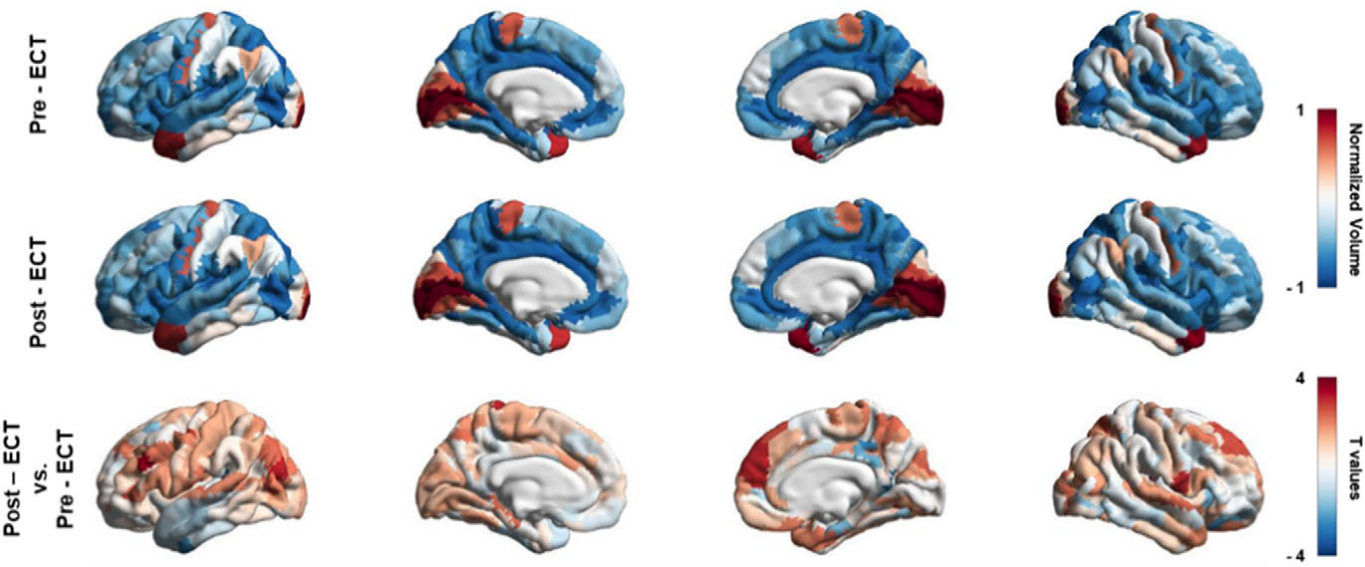
The above figure sequentially demonstrates the distribution of GMV before (top row) and after (middle row) ECT treatment, and differences in GMV before and after ECT treatment (bottom row). A positive (negative) *T*-value represents an increase (a decrease) of the GMV in this region after ECT treatment.

As shown in Figure 1, a positive (negative) T-value represents an increase (a decrease) of the GMV in this region after ECT treatment. After ECT treatment, GMV in the somatosensory and motor cortex, occipital cortex, prefrontal cortex, and insula showed a tendency to increase, while that in the temporal cortex, post-cingulate cortex and orbitofrontal cortex showed a tendency to decrease(Figure 1). To better illustrate the regional changes of GMV, we showed four regions that show significant changes after ECT treatment (Supplementary Figure 1). These suggest that ECT may achieve therapeutic effects by modulating the brain structure of patients.

### Clinical profiles related to GMV changes

**Figure 2.**
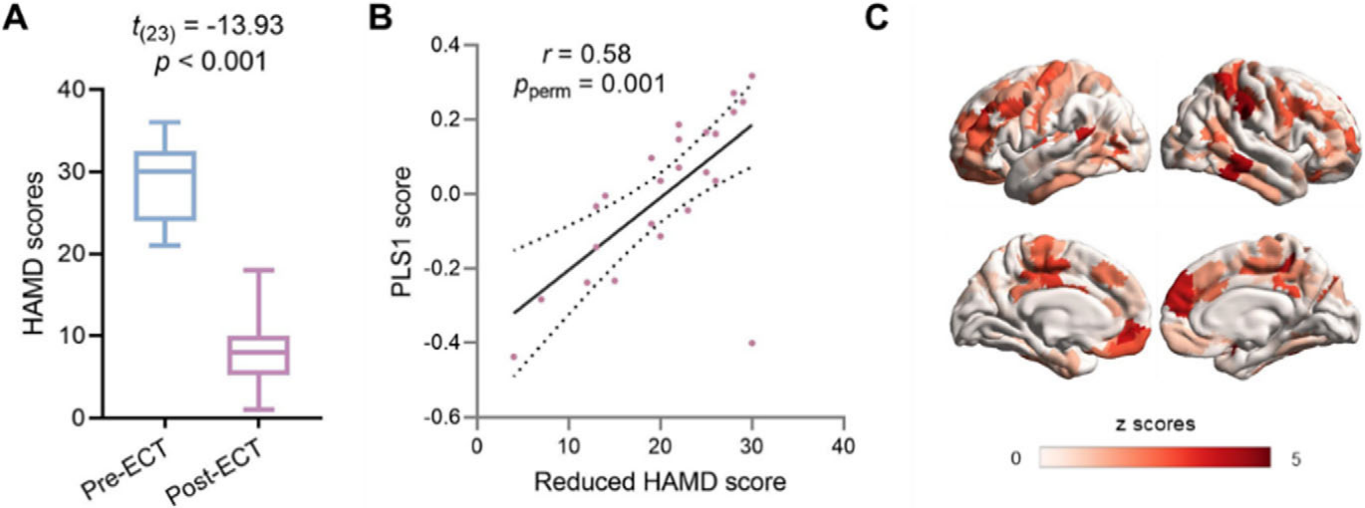
Clinical profiles related to GMV changes. (a) Differences in depressive symptoms before and after ECT treatment. The *t* value represents the paired *t*-test statistic (Post-ECT vs. Pre-ECT). (b) Correlation between PLS1 score and reduced HAMD score. (c) Regional contribution to the prediction, the *z*-score was defined as the ratio of the weight of each regional GMV to the standard error.

To reveal the antidepressant effect of ECT, depressive symptom was measured with HAMD. We found that HAMD scores decreased significantly after ECT treatment (t _(23)_ = −13.93, p < 0.001; Figure 2a). We further investigated the relation between improved depressive symptoms and changes in GMV with a PLS regression analysis. Differences in GMV before and after treatment were used as predictors for improved depressive symptoms, which were measured as reduced HAMD scores. GMV was a significant predictor of HAMD scores (*r* = 0.58, *p*_perm_ = 0.001), in addition, the visual cortex, IPC, and part of the insula showed higher weight for HAMD prediction (Figure 2b). Brain regions showing high contribution to HAMD score prediction were mainly located in the prefrontal cortex, post cingulate cortex, and the inferior temporal cortex (Figure 2c). These regions largely overlapped with regions showing ECT-induced changes in GMV.

### Cortical gene expression related to GMV changes

**Figure 3.**
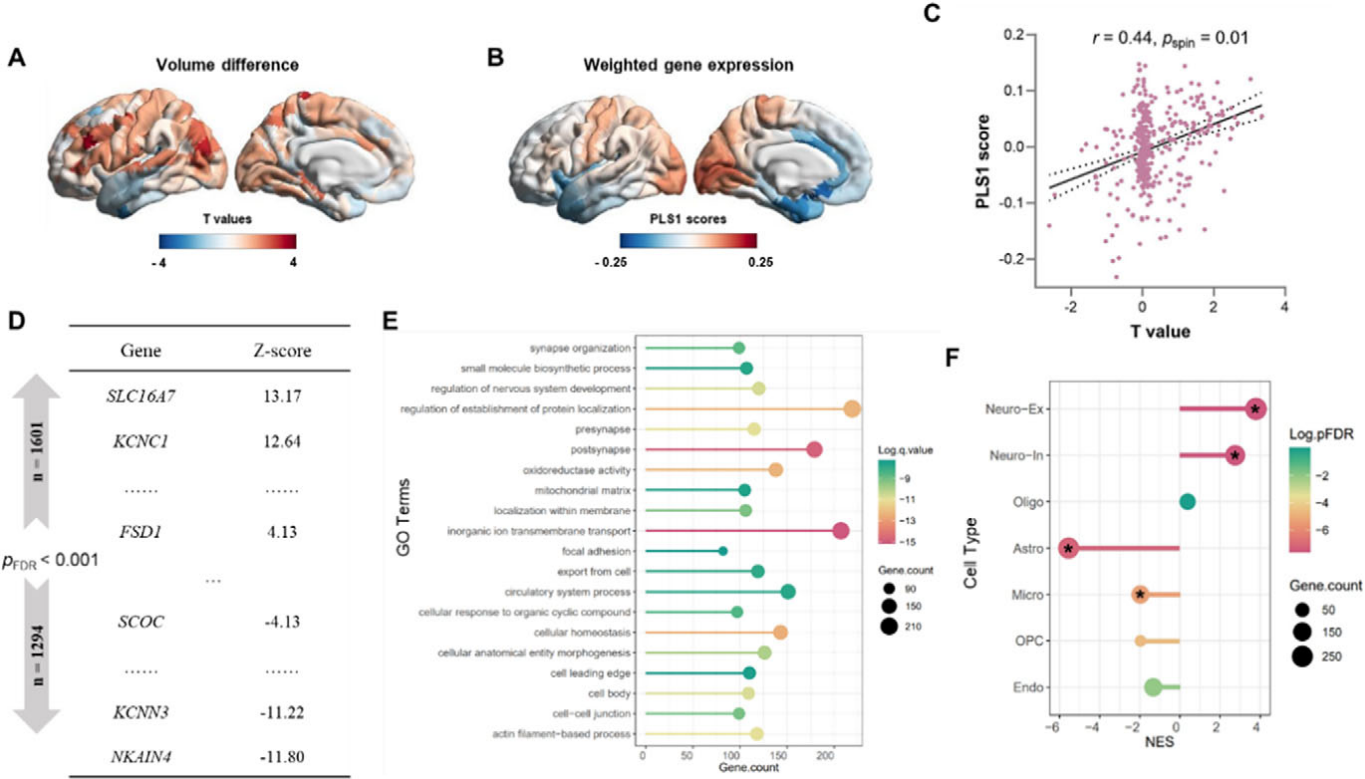
Functional pathways and cell types associated with changes in gray matter volume after ECT. (a) Changes in gray matter volume after ECT treatment (left hemisphere). (b) Gene weight expression map obtained by PLS regression analysis. (c) Correlation between gray matter volume change after ECT treatment and PLS1 gene weight combination. (d) Schematic diagram of the sorted gene list. (e) Gene enrichment pathway, where the size of the circle is the rich gene number in the pathway. (f) Gene enriched cell types.

A PLS regression analysis was performed to determine the patterns of gene expression that were correlated with changes of GMV. We found that PLS1 accounted for 19% of the variance in GMV changes, and the explanation rate was significant (*p*_spin_ = 0.013). The weight map distribution of PLS1 showed higher expression in somatosensory and motor cortex and occipital cortex, while lower expression in temporal cortex and orbitofrontal cortex (Figure 3b), and the weighted gene expression showed a significant positive spatial correlation with GMV changes (Figure 3c). Sequencing the genes by weight, we found that the expression patterns of 1601 PLS1+ genes and 1294 PLS1− genes were positively (or negatively) correlated with GMV changes (*p*_FDR_ < 0.001, Figure 3d). The positive correlations hint that PLS1+ genes are overexpressed in regions where GMV increases, while PLS1− genes are overexpressed in regions where GMV decreases.

### PLS1 genes enriched into specific functional pathways and cell types

Gene enrichment analysis was performed to investigate whether the PLS1 genes are involved in specific functional pathways. We found that PLS1 genes were enriched for synapse- and cell-related biological processes and cellular components, such as ‘pre- and post-synapse’, ‘synapse organization’, ‘cellular homeostasis’, ‘cellular anatomical entity morphogenesis’, ‘cell-cell junction’, ‘export from cell’, ‘focal adhesion’ and ‘cellular response to organic cyclic compound’ (Figure 3e). We further examined whether PLS1 genes are expressed in specific cell types. We found that a number of gene in the PLS1+ gene list was significantly involved in Excitatory neurons (Neuro-Ex) (number = 290, *p*_FDR_ < 0.01) and Inhibitory neurons (Neuro-In) (number = 213, *p*_FDR_ < 0.01), and the PLS1− genes were significantly involved in Astro (number = 252, *p*_FDR_ < 0.01) and Micro (number = 137, *p*_FDR_ < 0.01, Figure 3f). These findings linked gene expression associated with ECT-induced GMV changes to specific functional pathways and cell types, which advance the understanding of the underlying molecular mechanisms of ECT-induced brain structural changes.

## Discussion

This study attempts to gain a deeper understanding of the mechanisms of ECT through clinical research on patients with MDD, firstly explained the underlying mechanism of changes in GMV caused by eight ECT sessions using a combination of imaging and transcriptomics analysis. After bilateral-temporal ECT, the GMV in the insula, occipital, prefrontal, and somatosensory and motor cortices tended to increase, but the GMV of the temporal, posterior cingulate, and orbitofrontal cortices tended to decrease. Differences in GMV caused by ECT were significant predictors of variations in HAMD scores. However, only four brain regions (dorsolateral prefrontal cortex, inferior parietal cortex, anterior cingulate and medial prefrontal cortex, posterior opercular cortex) showed a significant increase in GMV. Furthermore, this study discovered that patterns of GMV variation induced by ECT in MDD were linked to synaptic and cell-associated pathways employing AHBA. Microglia and astrocytes mainly explained the GMV reduction caused by ECT., whereas neurons mainly contributed to the GMV increase. The understanding of the potential mechanism behind the antidepressant effects of ECT is advanced by these findings, which close the knowledge gap between transcriptome and neuroimaging.

Previous researches have revealed structural anomalies in the prefrontal lobe, precuneus, cingulate gyrus, para-hippocampus, temporal cortex, and some other depression-related regions (Bremner et al., 2000; Roberson-Nay et al., 2020; Sheline, 2000; Vythilingam et al., 2004). Although most regional changes were not significant, this study found that ECT can lead to increases or decreases in GMV across a wide range of brain areas, which may be related to the fact that ECT is a treatment that induces widespread brain discharge. ECT was reported to result in an increase in GMV in certain brain regions in patients with MDD (Enneking et al., 2020) most of those were in cortical temporal area, insula, anterior cingulate cortex, striatum, posterior central gyrus, fusiform gyrus, orbitofrontal cortex, and auxiliary motor system (Ota et al., 2015; Yamasaki et al., 2020; Zhou et al., 2024) including those identified in this study as showing GMV increases due to ECT. We did not find any significant changes in the volume of the hippocampus, which may be related to our small sample size and insufficient sensitivity to changes in GMV. Moreover, to further illustrate the relationship between the changes in GMV caused by ECT and its antidepressant effects, this study have correlated the change in GMV with the rate of HAMD score reduction. And we found GMV changes in prefrontal cortex, post-cingulate cortex, and the inferior temporal cortex showed high contribution to HAMD score prediction. These brain regions are believed to play an important role in the pathological mechanisms of depression, and are closely connected to the hippocampus both structurally and functionally (Dukart et al., 2014; Dziobek et al., 2011; Foster et al., 2023; Fox et al., 2006; Jorgensen et al., 2016; Li et al., 2013; Rolls, Cheng, & Feng, 2020).

Human genetic imaging has been a potent tool recently for deciphering the molecular causes of variations in neuroimaging (Fornito et al., 2019; Morgan et al., 2019; Romero-Garcia et al., 2020; Romme et al., 2017) Previous functional enrichment analyses have demonstrated some MDD-associated and ECT-associated genes, the majority of which are involved in drug response processes, metabolic functions, immunological system, cell signaling, and neurodevelopment (Fan et al., 2020). This study found ECT-induced GMV changes were associated with GO bio-enrichment pathways (‘presynaptic and postsynaptic’, ‘synaptic organization’, ‘cell homeostasis’, ‘cell anatomical entity morphogenesis’, ‘cell-cell connectivity’, ‘cell output’, ‘focal adhesion’, and ‘cell response to organic cyclic compounds’), which regulate multiple synaptic transmission or neurological functions. These pathways are basically consistent with previous findings. The above findings can be supported by animal studies and postmortem research (Duman & Aghajanian, 2012; Fan et al., 2020). Previous research has shown that depressive behavior in rodents could be caused by synaptic impairments. (Kang et al., 2012). Similar findings were reported for postmortem dendritic morphology in patients with depression (J. Li et al., 2021). Furthermore, ECT was thought to be mediated hippocampal neuroplasticity which linked to both cognitive function and antidepressant effects (Gbyl et al., 2021a, 2021b). This study found that both excitatory and inhibitory neurons were linked to ECT-induced GMV increase in patients with MDD, which is in line with the results of a single-nucleus transcriptomics research of depression (Yirmiya, Rimmerman, & Reshef, 2015). On one hand, ECT could directly activate excitatory neurons through electrical stimulation, leading to widespread neural discharge and seizure-like activity, which in turn promotes the expression of genes related to neural plasticity, enhances synaptogenesis and neuronal connectivity, resulting in an increase in GMV (Duman & Aghajanian, 2012). On the other hand, the activation of inhibitory neurons by ECT may help balance excitatory activity, preventing neuronal damage caused by excessive excitation, and promoting synaptic plasticity changes (Kang et al., 2012), thereby increasing the volume and density of gray matter. In addition, numerous neuroimaging techniques have frequently shown a substantial correlation between astrocytes (Zhou et al., 2024) or microglia’s activation (Dukart et al., 2014) and pathological abnormalities in depression. Astrocytes and microglia are both present in the gray matter. The former provides nutrients and structural support to neurons and participates in synaptic plasticity, while the latter serves as the main immune cells in the central nervous system, playing roles in immune surveillance, synaptic remodeling, and regulating inflammatory responses. The current study showed that astrocytes and microglia had a disproportionately high expression of genes related to GMV decreases in the temporal cortex, post-cingulate cortex, and orbitofrontal cortex produced by ECT. This finding is consistent with earlier findings (Zhou et al., 2024), and possibly related to the following mechanisms. First, the two types of glial cells might activate inflammatory responses, oxidative stress, and the formation of glial scars, leading to neuronal damage (Jorgensen et al., 2016), which may reveal the cognitive side effects of ECT. Second, glial cells could involve synaptic pruning and remodeling, promoting synaptic functional stability (Dziobek et al., 2011), which may explain the efficacy of ECT.

There are still certain limitations that suggest further research should pay attention to. First, the sample size for pathway enrichment analysis brain imaging analysis in this study is relatively small, which may affect the reliability and stability of the results. Second, the gene expression data used in this work were obtained from post-mortem brain tissues without a psychiatric diagnosis, and were only from the left side of the brain. This may have limited the analysis of transcriptome–neuroimaging associations. Third, as an observational study, we did not control for medication use during ECT. Most patients were concurrently using antidepressants, and although the effects of the medication may have been limited over ~2 weeks, they could still influence clinical efficacy and brain imaging. Therefore, the results of this study can only be considered a preliminary indication of the mechanisms of ECT. Future research should encompass the entire brain for imaging analysis, use larger sample size and observe patients with depression treated solely with ECT.

## Conclusion

This study has made a certain contribution in understanding the therapeutic mechanisms of ECT. It explored GMV changes in MDD patients and further analyzed the possible underlying functional pathways and cell types that may explain the ECT-induced changes of brain structure, which links biological with imaging mechanisms in clinical patients with MDD and provides further evidence for the antidepressant mechanisms of ECT.

## Supporting information

Liu et al. supplementary material Liu et al. supplementary material

## Supplementary material

The supplementary material for this article can be found at https://doi.org/10.1017/S0033291725000868.
