# Supplementary material for: Alterations in gray matter volume and associated transcriptomics after electroconvulsive therapy in major depressive disorder
Source: Psychol Med. 2025 Apr 21;55:e118. doi: 10.1017/S0033291725000868 (PMC12094620; doi:10.1017/S0033291725000868)
Supplement: Liu et al. supplementary material [file S0033291725000868sup001.docx]

# Supplementary Material


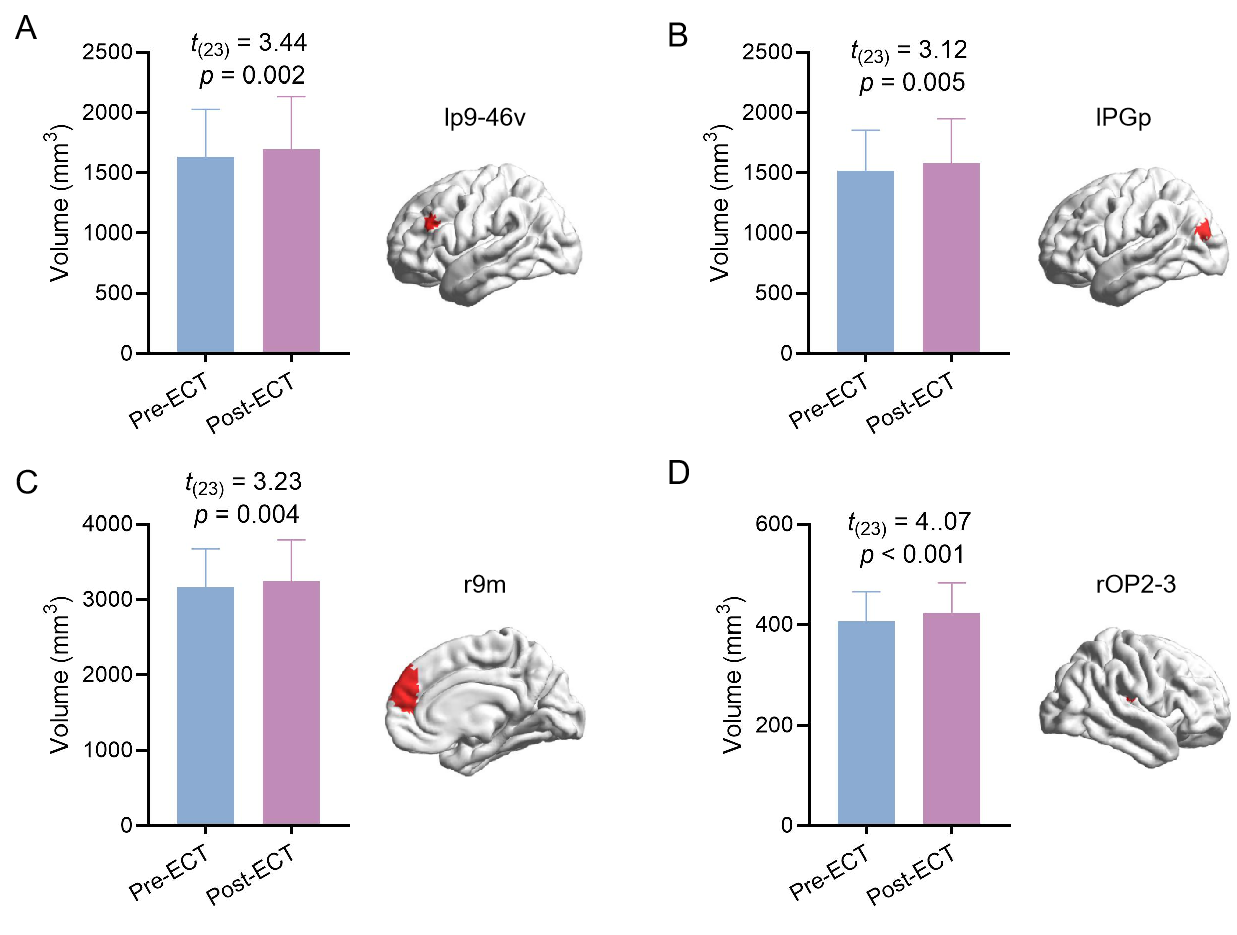


Supplementary Figure 1. Regional differences in GMV before and after ECT treatment. The *t* values represent the paired t-test statistic (Posr-ECT vs. Pre-ECT). lp9-46v, left area posterior 9-46v (dorsolateral prefrontal cortex); lPGp, left area PGp (inferior parietal cortex); r9m, right area 9 middle (anterior cingulate and medial prefrontal cortex); rOP2-3, right area OP2-3/VS, (posterior opercular cortex).
